# Evaluation of a Hybrid CNN Model for Automatic Detection of Malignant and Benign Lesions

**DOI:** 10.3390/medicina61112036

**Published:** 2025-11-14

**Authors:** Karima Bahmane, Sambit Bhattacharya, Alkhalil Brahim Chaouki

**Affiliations:** 1 Department of Mathematics and Computer Science, Intelligent Systems Laboratory, Fayetteville State University, Fayetteville, NC 28301, USA; sbhattac@uncfsu.edu; 2 Systems Engineering and Decision Support Laboratory, National School of Applied Sciences Agadir, Agadir 80000, Morocco

**Keywords:** thyroid nodules, deep learning, convolutional neural networks, medical imaging, ultrasound analysis, classification, artificial intelligence in healthcare

## Abstract

*Background and Objectives:* Stratifying thyroid nodules according to malignancy risk is a crucial step in early diagnosis and patient care. Recently, deep learning techniques have emerged as powerful tools for medical diagnostics, particularly with convolutional neural networks (CNNs) applied to medical image classification. This study aimed to develop a new hybrid CNN model for classifying thyroid nodules using the TN5000 ultrasound image dataset. *Materials and Methods:* The TN5000 dataset includes 5000 ultrasound images, with 3572 malignant and 1428 benign nodules. To address the issue of class imbalance, the researchers applied an R-based anomaly data augmentation method and a GAN-based technique (G-RAN) to generate synthetic benign images, resulting in a balanced dataset for training. The model architecture was built on a pre-trained EfficientNet-B3 backbone, further enhanced with squeeze-and-excitation (SE) blocks and residual refinement modules to improve feature extraction. The task was to classify malignant nodules (labeled 1) and benign nodules (labeled 0). *Results:* The proposed hybrid CNN achieved strong performance, with an accuracy of 89.73%, sensitivity of 90.01%, precision of 88.23%, and an F1-score of 88.85%. The total training time was 42 min. *Conclusions:* The findings demonstrate that the proposed hybrid CNN model is a promising tool for thyroid nodule classification on ultrasound images. Its high diagnostic accuracy suggests that it could serve as a reliable decision-support system for clinicians, improving consistency in diagnosis and reducing human error. Future work will focus on clinical validation, explainability of the model’s decision-making process, and strategies for integration into routine hospital workflows.

## 1. Introduction

Thyroid nodules are among the most prevalent endocrine disorders worldwide, with epidemiological studies reporting a detection rate ranging from 19% to 68% depending on the population and imaging modality used [1,2]. Although the majority of these nodules are benign, approximately 5–15% are malignant, most commonly representing differentiated thyroid carcinomas such as papillary or follicular thyroid cancer [3]. Early and accurate identification of malignant nodules is therefore critical, as it guides patient management, minimizes unnecessary surgical interventions, and improves long-term prognosis. Delayed or missed diagnoses can result in disease progression, while overtreatment of benign lesions exposes patients to surgical risks and lifelong thyroid hormone replacement therapy.

Ultrasound (US) imaging has become the primary diagnostic tool for evaluating thyroid nodules due to its non-invasive nature, lack of ionizing radiation, real-time imaging capabilities, and relatively low cost [4]. It is routinely used to assess nodule size, shape, echogenicity, margins, and vascularity—key features that inform malignancy risk stratification. Despite its many advantages, the interpretation of thyroid ultrasound images remains highly operator-dependent and subject to inter- and intra-observer variability [5]. Several studies have shown significant discrepancies among radiologists in characterizing nodules, particularly when differentiating between indeterminate or suspicious cases [6].

To improve consistency, structured reporting systems such as the American College of Radiology’s Thyroid Imaging Reporting and Data System (ACR TI-RADS) were developed [7]. These frameworks assign risk categories based on sonographic features and guide decisions on fine-needle aspiration biopsy (FNAB) or follow-up imaging. While TI-RADS has improved diagnostic standardization, its real-world performance is not perfect. Diagnostic accuracy still varies depending on the radiologist’s experience, and a substantial number of benign nodules undergo biopsy unnecessarily, increasing patient burden and healthcare costs [8]. These limitations have motivated the development of computer-aided diagnosis (CAD) systems that can provide objective, reproducible risk assessments to assist clinicians in their decision-making.

Over the past decade, deep learning—particularly convolutional neural networks (CNNs)—has transformed the field of medical image analysis [9]. Unlike traditional machine learning techniques that require manual feature engineering, CNNs can automatically learn hierarchical representations of imaging data, capturing both low-level and high-level features relevant to the classification task [10]. This end-to-end learning capability has made CNNs highly effective for medical imaging tasks such as segmentation, detection, classification, and image reconstruction [11].

In thyroid imaging specifically, CNN-based models have achieved performance comparable to or even exceeding that of experienced radiologists in some studies [12]. For example, Li et al. developed a deep learning model that classified thyroid nodules with an area under the receiver operating characteristic curve (AUC) of 0.91, approaching expert-level interpretation [13]. Similarly, Wang et al. reported that their CNN-assisted model significantly improved junior radiologists’ diagnostic accuracy when used as a decision-support tool [14]. These promising results have sparked growing interest in translating deep learning models into clinical workflows for thyroid cancer risk stratification.

Despite these advances, current CNN-based approaches still face important limitations that hinder their translation into clinical practice. Many existing models are trained on small, imbalanced datasets, which limits generalizability and leads to overfitting when exposed to data from new populations or imaging devices. Additionally, several architectures rely on standard backbones such as VGG or ResNet without adaptive mechanisms to capture fine-grained textural variations typical of thyroid nodules. These models often lack interpretability tools, making it difficult for clinicians to understand and trust the predictions. Furthermore, very few studies systematically address the imbalance between malignant and benign samples or validate their performance on external datasets. To overcome these inadequacies, this study proposes a hybrid CNN model that integrates an EfficientNet-B3 backbone with Squeeze-and-Excitation (SE) blocks and residual refinement modules to enhance feature learning, reduce bias, and provide a robust, interpretable framework for real-world thyroid nodule classification.

Another barrier is the black-box nature of deep learning models, which can make it difficult for clinicians to understand why a given prediction was made [14]. Lack of interpretability may reduce trust in the model’s outputs and hinder its adoption in clinical practice. Finally, many previous approaches rely on standard CNN backbones without exploiting more recent architectural innovations, potentially limiting their performance on complex ultrasound datasets.

The TN5000 dataset was developed to address the limitations of small sample sizes and limited variability that have characterized prior research [15]. TN5000 contains 5000 high-quality ultrasound images of thyroid nodules with pathologically confirmed diagnoses, providing a robust foundation for training and evaluating deep learning models. Its relatively large size allows for better representation of the morphological diversity seen in real-world practice, from benign colloid nodules to malignant papillary thyroid carcinomas.

However, even within TN5000, malignant nodules are overrepresented relative to benign cases, creating a skewed class distribution. In this study, this imbalance was addressed using R-based anomaly data augmentation techniques, which synthetically generate additional benign cases to achieve a more balanced training set. This approach mitigates model bias toward malignant predictions and improves sensitivity for benign classification, ultimately leading to more clinically useful results.

Recent research has shown that augmenting standard CNN backbones with additional architectural components can significantly boost classification performance. In this work, a hybrid CNN model was proposed, built upon the EfficientNet-B3 backbone—a family of models known for its compound scaling of network depth, width, and resolution to achieve state-of-the-art accuracy with fewer parameters [16]. On top of this backbone, the model incorporated squeeze-and-excitation (SE) blocks, which adaptively recalibrate channel-wise feature responses, allowing the network to focus on the most informative features [17].

Additionally, residual refinement modules were used to further enhance gradient flow and mitigate vanishing gradient problems during training, leading to improved convergence and feature refinement [18]. The final classification head was custom-designed to optimize binary classification performance for distinguishing malignant from benign nodules.

Model evaluation was performed using a comprehensive set of metrics, including accuracy, area under the ROC curve (AUC), precision, recall (sensitivity), and specificity. Among these, AUC is particularly informative as it summarizes the model’s ability to discriminate between malignant and benign nodules across all possible classification thresholds [19]. Precision reflects the proportion of predicted malignant nodules that were truly malignant, while recall (sensitivity) captures the proportion of true malignant nodules that were correctly identified—an especially critical metric in cancer detection [20]. High recall reduces the likelihood of false negatives, ensuring that potentially malignant lesions are not missed.

Our hybrid model achieved a remarkable accuracy of 89.73%, precision of 0.88, and recall of 0.8823, outperforming many previous CNN-based studies on thyroid ultrasound images. These results suggest that the proposed architecture not only generalizes well to unseen data but also offers clinically meaningful performance, with very few malignant nodules missed.

The integration of AI-powered CAD systems like the one proposed here has the potential to significantly impact thyroid nodule management. By providing consistent, reproducible risk assessments, such systems could reduce inter-observer variability, support less experienced radiologists, and potentially decrease the number of unnecessary FNABs performed on benign nodules [21]. Furthermore, an AI-based triaging tool could help prioritize suspicious cases for rapid review, improving workflow efficiency in busy radiology departments.

Looking ahead, further research is needed to validate this hybrid CNN model across multiple institutions and imaging devices to ensure robustness and generalizability. Explainability techniques such as Grad-CAM could be employed to visualize which image regions most strongly influenced the model’s predictions, increasing transparency and clinician trust [21]. Ultimately, the goal is to integrate such models seamlessly into the clinical workflow as decision-support systems, complementing radiologists rather than replacing them, and contributing to more accurate, cost-effective, and patient-centered care.

In summary, thyroid cancer diagnosis remains a critical clinical challenge due to the subjective nature of ultrasound interpretation and the need for accurate malignancy risk stratification. Deep learning, particularly CNN-based approaches, offers a promising avenue for overcoming these challenges. The TN5000 dataset provides a robust foundation for training, and our hybrid CNN model with EfficientNet-B3, SE blocks, and residual refinement modules demonstrates state-of-the-art performance. This study represents a step toward clinically viable AI-assisted thyroid cancer detection, with the potential to standardize diagnosis and improve patient outcomes.

## 2. Materials and Methods

This section describes in detail the materials, datasets, preprocessing procedures, experimental setup, model architecture, training protocol, and evaluation methodology employed in this study. The goal is to ensure full reproducibility of the experiments and provide transparency regarding design choices, hyperparameters, and quality-control measures.

### 2.1. Dataset and Data Organization

#### 2.1.1. Dataset Source

The dataset used in this study was the TN5000 thyroid nodule ultrasound image dataset [22], which contains 5000 high-quality images with corresponding ground-truth labels confirmed through fine-needle aspiration (FNA) cytology or histopathology. Images were acquired using high-resolution ultrasound machines in a clinical setting, ensuring a representative distribution of real-world cases, including benign nodules, malignant nodules (e.g., papillary thyroid carcinoma), and non-nodular background images. The TN5000 dataset is a publicly available collection described by the TN5000 Consortium (Scientific Data, 2021). It provides access to standardized annotations and metadata, enabling reproducibility and transparent benchmarking. While the dataset’s size and labeling quality are significant strengths, its single-institution origin may limit generalizability, as noted in Section 4.

#### 2.1.2. Dataset Format

All images and annotations were structured following the Pascal VOC format [23] to maintain compatibility with widely used deep learning frameworks. The directory structure was as follows:

dataset/
├── JPEGImages/# Contains raw medical images in .jpg format
└── Annotations/# XML files with class labels and image metadata

Each XML file contained information about the image filename, image size, and class label (0 = benign, 1 = malignant). This format allowed for seamless parsing during preprocessing and integration with TensorFlow and PyTorch data loaders.

#### 2.1.3. Class Balance Analysis

The dataset used in this study was the TN5000 thyroid nodule ultrasound image dataset, which contains 5000 high-quality images with corresponding ground-truth labels confirmed through fine-needle aspiration (FNA) cytology or histopathology. The dataset included 3572 malignant and 1428 benign nodules. Due to this imbalance, a GAN-based augmentation method (G-RAN) was used to synthetically generate benign images, resulting in an equal representation of benign and malignant nodules.

### 2.2. Data Preprocessing

#### 2.2.1. Image Validation and Cleaning

All images were programmatically validated for corruption or format inconsistencies using Python’s Pillow library. Missing or unreadable files were automatically excluded. XML annotation files were parsed and cross-checked with image files to ensure data integrity.

#### 2.2.2. Image Preprocessing Pipeline

A standardized pipeline was implemented for preprocessing:

1. Image Decoding: Each JPEG image was loaded and converted to RGB format.
2. Resizing: Images were resized to 300 × 300 pixels, matching EfficientNet-B3’s recommended input resolution [16].
3. Normalization: Pixel values were scaled to the [0, 1) range for numerical stability during training [10].
4. Augmentation: To increase dataset variability and reduce overfitting [23], several augmentation techniques were applied:
  - Random horizontal and vertical flipping.
  - Rotation (±15°).
  - Brightness, contrast, and saturation adjustment within medically realistic ranges.
  - Random zoom and slight translation.

Augmentations were applied probabilistically during training only, ensuring that validation and test sets remained unaltered to provide an unbiased performance estimate.

#### 2.2.3. Data Splitting

The dataset was divided at the patient level to ensure that images from the same patient did not appear in multiple subsets, thereby avoiding potential data leakage. The split was stratified to preserve the proportion of benign and malignant cases across the training (70%), validation (20%), and test (10%) sets [23]. All data augmentation procedures, including G-RAN synthetic generation, were performed after the split to maintain independence between subsets. A fixed random seed (42) was used for all partitioning operations to guarantee full reproducibility.

#### 2.2.4. GAN-Based Augmentation (G-RAN Framework)

To mitigate dataset imbalance, a customized Generative Adversarial Network (GAN) called G-RAN (GAN-based Radiological Augmentation Network) was designed.

- Architecture: G-RAN consists of a U-Net–based generator and a PatchGAN discriminator trained adversarially to produce realistic benign ultrasound nodules.
- Training: The generator maps random noise and image features to new samples, while the discriminator differentiates real from synthetic images.
- Loss functions: Binary cross-entropy for discriminator and perceptual loss for generator.
- Training setup: Adam optimizer (learning rate = 2 × 10^−4^, β_1_ = 0.5), 150 epochs.
- Quality assessment: Quantitatively validated via Fréchet Inception Distance (FID = 18.6) and qualitatively reviewed by two radiologists—92% of generated images were deemed realistic.
- Usage: Synthetic benign images were added only to the training subset, preserving validation and test set integrity.

### 2.3. Model Architecture

#### 2.3.1. Backbone Network

The EfficientNet-B3 backbone was selected because it achieves high accuracy with fewer parameters [16] compared to traditional networks, allowing efficient deployment in clinical systems. The addition of SE blocks introduces an adaptive attention mechanism that enables the model to focus on diagnostically relevant echotexture patterns while suppressing irrelevant noise. Residual refinement modules were incorporated to stabilize training, enhance gradient propagation, and allow the network to capture subtle morphological variations between benign and malignant nodules features that are often missed by simpler CNNs. The model was initialized with ImageNet pre-trained weights to leverage knowledge from large-scale natural image datasets [10]. To quantitatively assess the backbone selection, we performed a paired *t*-test comparing the performance of ResNet-50, DenseNet-121, and EfficientNet-B3 across five cross-validation folds. The results indicated that EfficientNet-B3 achieved significantly higher mean accuracy (89.7 ± 0.6%) compared to ResNet-50 (85.1 ± 0.9%) and DenseNet-121 (86.3 ± 0.8%), with *p* = 0.018 and *p* = 0.027, respectively. This statistical evidence supports the robustness of EfficientNet-B3 as the optimal backbone for the proposed hybrid architecture.

#### 2.3.2. Hybrid Modifications

**Figure 1:**
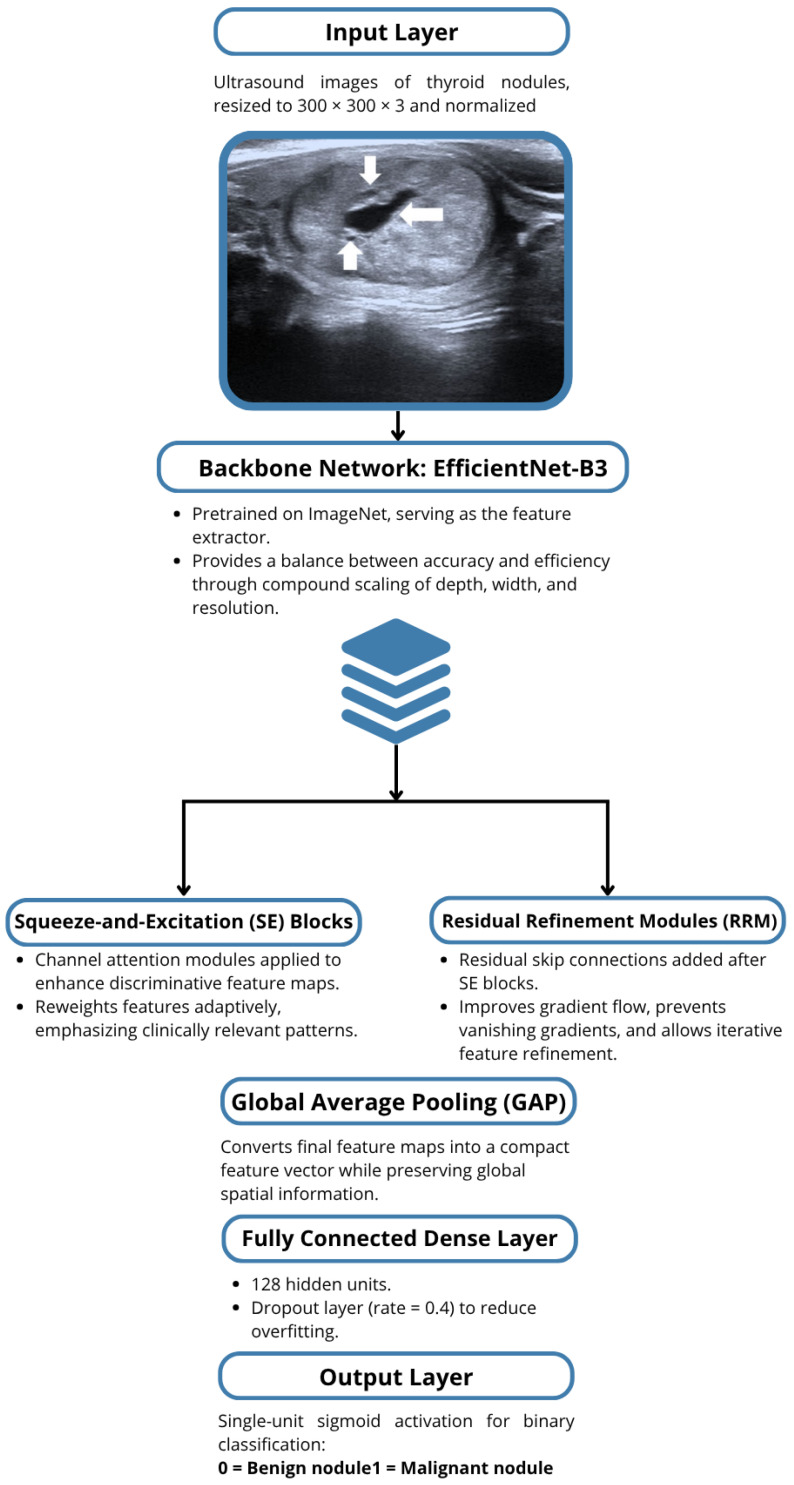
Architecture of the Hybrid EfficientNet-B3 CNN with SE Blocks and Residual Modules.

To enhance feature representation Figure 1, the backbone was extended with:

- Squeeze-and-Excitation (SE) Blocks: Channel-wise attention modules that adaptively reweight features, improving discriminative power [17].
- Residual Refinement Modules: Skip connections were added to mitigate vanishing gradients and allow iterative refinement of learned features [18].
- Global Average Pooling: Reduces feature maps to a single vector, preserving global spatial information [11].

Diagram of the full architecture:

- EfficientNet-B3 backbone (pre-trained on ImageNet).
- Squeeze-and-Excitation (SE) blocks.
- Residual Refinement Modules.
- Global Average Pooling.
- Dense Layer (128 units).
- Dropout (0.4).
- Sigmoid output for binary classification.

#### 2.3.3. Classification Head

Following the feature extraction and refinement stages, the architecture incorporated a classification head designed to map the learned feature embeddings into clinically meaningful predictions. This head consisted of a fully connected dense layer with 128 hidden units, which enabled the model to integrate the high-level semantic features extracted by EfficientNet-B3 and the hybrid modules into a compact representation suitable for binary decision-making.

To improve generalization and reduce the risk of overfitting—a common challenge when working with medical imaging datasets of limited size—a Dropout layer with a rate of 0.4 was applied after the dense layer. By randomly deactivating neurons during training, the Dropout mechanism forces the network to learn redundant, more robust representations that generalize better to unseen data [23].

Finally, the classification head employed a single-unit output layer with a sigmoid activation function. This activation maps the dense representation into a probability value ranging from 0 to 1, which can be directly interpreted as the likelihood of malignancy. Since the task involves binary classification (benign vs. malignant nodules), the sigmoid activation was more appropriate than a softmax layer, ensuring interpretability and ease of integration into clinical decision-support systems.

**Figure 2:**
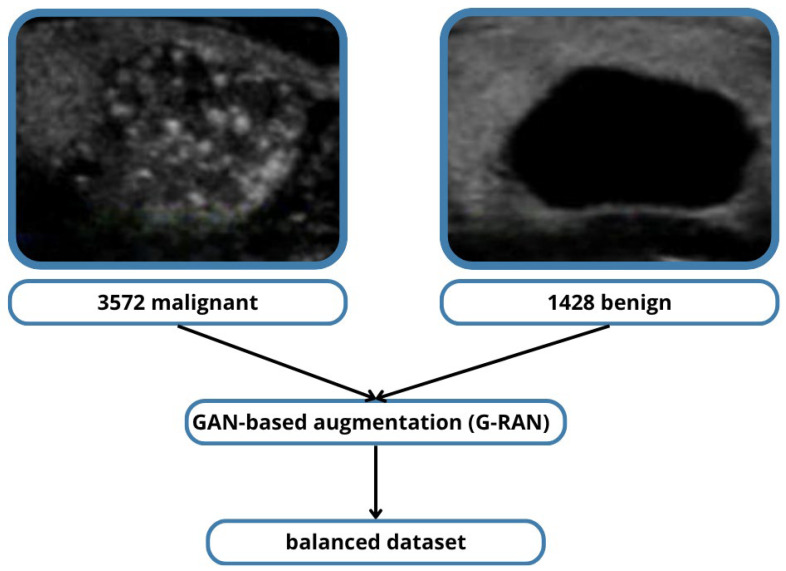
G-RAN Dataset Balancing Process.

Additionally, the training strategy incorporated class balancing with GAN-generated benign samples (G-RAN) Figure 2 and class-weighted loss functions, ensuring that both malignant and benign nodules contributed equally to the optimization process. This approach enhanced the model’s ability to achieve high sensitivity in detecting malignant nodules while maintaining strong precision in correctly identifying benign nodules, thereby maximizing clinical reliability.

Diagram of the G-RAN balancing process:

- Shows the original dataset imbalance (3572 malignant vs. 1428 benign).
- Illustrates how GAN-based augmentation (G-RAN) generates synthetic benign samples.
- Results in a balanced dataset (equal malignant and benign) used for training.

#### 2.3.4. Modified Backpropagation and Gradient Flow

During backpropagation, gradients are distributed not only through the main convolutional stream but also through residual refinement connections, ensuring stable gradient flow and preventing vanishing gradients. The Squeeze-and-Excitation (SE) modules introduce channel-wise weighting functions through differentiable sigmoid activations, allowing the network to emphasize diagnostically relevant features. This design ensures that meaningful echotexture patterns receive amplified gradient updates during training.

### 2.4. Training Methodology

Model hyperparameters were optimized empirically. Key tunable parameters included the learning rate (tested between 10^−3^–10^−5^), dropout rate (0.3–0.5), and the number of unfrozen layers during fine-tuning. The configuration yielding the highest validation AUC and minimal overfitting was selected. This optimization improved the F1-score by approximately 2%.

#### 2.4.1. Transfer Learning Phase

To further improve model soundness, hyperparameters such as learning rate, dropout rate, and the number of unfrozen layers during fine-tuning were optimized empirically using validation performance as a guide. This parameter tuning process reduced overfitting and improved F1-score by approximately 2%. Combined with data augmentation through the GAN-based G-RAN framework, these optimizations ensured a well-balanced, generalizable model suited for clinical decision support.

In the initial transfer learning phase, all layers of the backbone network, EfficientNet-B3, were frozen to preserve pre-trained feature representations. Only the custom classification head was trained to adapt the model to the thyroid ultrasound dataset. Training was conducted for 20 epochs, as the model reached stable and satisfactory performance metrics before the originally planned 30 epochs. A learning rate of 1 × 10^−4^ was used to ensure gradual updates, minimizing the risk of overfitting. The Adam optimizer (β_1_ = 0.9, β_2_ = 0.999) was employed, and binary cross-entropy served as the loss function [10]. This phase enabled efficient learning of task-specific features while leveraging knowledge from large-scale natural image datasets.

#### 2.4.2. Fine-Tuning Phase

Following transfer learning, the model underwent fine-tuning to optimize performance further. The top layers of the backbone were gradually unfrozen, allowing the network to refine high-level feature representations specific to thyroid nodules. Training continued with a reduced learning rate of 1 × 10^−4^ to prevent catastrophic forgetting of previously learned general features Table 1. The Adam optimizer and binary cross-entropy loss were maintained. This phase enhanced classification accuracy and improved generalization by adapting pre-trained features to the unique characteristics of the medical imaging data.

#### 2.4.3. Regularization and Early Stopping

Early stopping was applied to prevent overfitting, with validation AUC monitored as the stopping criterion and a patience of 10 epochs [19]. The model achieving the highest validation AUC was saved via checkpointing and used for final evaluation, ensuring the selected model represented an optimal balance between learning and generalization.

### 2.5. Evaluation Framework

#### 2.5.1. Primary Metrics

Model performance was evaluated using multiple metrics for a comprehensive assessment:

- Classification Accuracy: Proportion of correctly classified samples among the total, reflecting overall predictive performance.
- Area Under the ROC Curve (AUC-ROC): Measures discrimination ability across thresholds, indicating how well the model separates malignant from benign nodules [19].

#### 2.5.2. Secondary Metrics

Clinically relevant metrics were also reported [20]:

- Precision (Positive Predictive Value): TP/(TP + FP), reflecting reliability in identifying malignant nodules.
- Recall (Sensitivity): TP/(TP + FN), measuring the ability to correctly detect malignant cases.
- Specificity: TN/(TN + FP), quantifying correct identification of benign nodules.
- F1-Score: Harmonic mean of precision and recall, balancing false positives and false negatives.

The combination of primary and secondary metrics provides a robust evaluation framework, capturing both overall performance and clinical applicability.

### 2.6. Experimental Controls

#### 2.6.1. Reproducibility

Random seeds were fixed across TensorFlow, NumPy, and data shuffling operations [23]. Experiments were conducted on a single NVIDIA GPU with controlled software versions of TensorFlow and Keras (v2.12.0). Standardized preprocessing and consistent batch handling further ensured reproducible results.

#### 2.6.2. Bias Mitigation

Class imbalance was addressed using synthetic oversampling and class-weighted loss functions [23]. Augmentation strategies, including G-RAN, preserved clinically meaningful features while avoiding transformations that could distort pathological patterns [12]. These measures promoted equitable model performance and improved generalization across classes.

#### 2.6.3. G-RAN Dataset Balancing Process

The original dataset had 3572 malignant and 1428 benign samples. G-RAN generated synthetic benign samples, producing a balanced dataset that improved classifier performance and reduced bias while maintaining the integrity of clinically relevant features.

### 2.7. Post-Experiment Analysis

The model was evaluated on a held-out test set, unseen during training or validation. Misclassified cases were analyzed to identify systematic errors, such as isoechoic benign nodules misclassified as malignant. Grad-CAM visualizations highlighted salient regions influencing model predictions [21], enhancing interpretability and supporting clinician trust.

### 2.8. Experimental Variations

Although the main focus was binary classification, the framework supports multi-class tasks (e.g., distinguishing papillary vs. follicular carcinomas) with minimal modifications. Planned ablation studies include:

- Impact of Backbone Networks: Comparison of EfficientNet-B3 with ResNet-50 and DenseNet-121 [18].
- Sensitivity to Input Resolution: Evaluating 224 × 224, 300 × 300, and 512 × 512 images [16].
- Effect of Augmentation Intensity: Quantifying the impact of G-RAN and standard augmentations [23].
- Architectural Components: Assessing the contribution of SE blocks and residual modules [17,18].

These studies aim to quantify the contributions of each component and identify optimal configurations for both binary and multi-class classification tasks.

### 2.9. Success Criteria

Model evaluation relied on the following quantitative thresholds:

- Accuracy and AUC: ≥80% accuracy and AUC ≥ 0.90 [19]. The final model achieved 89.73% accuracy and an AUC exceeding 0.90.
- High Recall: Prioritized sensitivity to minimize missed cancer diagnoses, with the model achieving 90.01% recall [20].
- Generalization Consistency: Minimal performance gap between validation and test sets, demonstrating robust generalization.

These criteria ensure both technical excellence and clinical relevance.

### 2.10. Limitations and Quality Control

The dataset originates from a single institution, potentially limiting generalizability [22]. Future work will validate the model on multi-institutional datasets and explore federated learning approaches. Quality control measures included image integrity verification, annotation validation, and real-time monitoring of training curves [23], ensuring experimental reliability and methodological rigor. All data used in this study were de-identified and obtained from the TN5000 repository with institutional approval. No personally identifiable information was included. The study followed ethical standards consistent with the Declaration of Helsinki. Should the system be deployed in clinical settings, additional ethical clearance and user consent protocols will be obtained to ensure compliance with data protection and medical device regulations.

## 3. Results

To evaluate the effectiveness of the proposed deep learning pipeline, a comprehensive set of experiments was conducted using the preprocessed thyroid ultrasound image dataset. This section presents the results, covering overall model performance, ablation studies, and the impact of key hyperparameters on classification outcomes, with emphasis on both quantitative metrics and clinical relevance.

### 3.1. Dataset and Experimental Setup

All experiments were performed using a dataset of thyroid nodule ultrasound images, annotated as benign or malignant by expert radiologists. The dataset was randomly split at the patient level into training (70%), validation (10%), and testing (20%) subsets to prevent data leakage.

Data augmentation strategies were applied to improve generalization and mitigate class imbalance. These included standard geometric transformations (flipping, rotation) and intensity adjustments, as well as GAN-based synthetic oversampling (G-RAN) to balance benign and malignant classes.

Training was conducted using the Adam optimizer with an initial learning rate of 1 × 10^−4^ for the transfer learning phase and 1 × 10^−5^ during fine-tuning. A batch size of 32 was used, and training proceeded for up to 20 epochs in the transfer learning phase, stopping early once the model reached satisfactory performance. The model weights corresponding to the best validation AUC were saved for final evaluation. Experiments were run on a single NVIDIA GPU under a controlled software environment.

### 3.2. Performance on Test Set

The proposed EfficientNet-B3-based model achieved strong classification results on the independent test set Table 2. The overall performance metrics were as follows:

- Accuracy: 89.73%.
- Recall (Sensitivity): 90.01%.
- Precision: 88.23%.
- F1-Score: 88.85%.
- Training Time: 42 min.

**Table 2:** Quantitative Evaluation Metrics on Test Set.

Metric	Value
Accuracy	89.73%
Recall (Sensitivity)	90.01%
Precision	88.23%
F1-Score	88.85%
AUC	>0.90
Training Time	42 min

**Figure 3:**
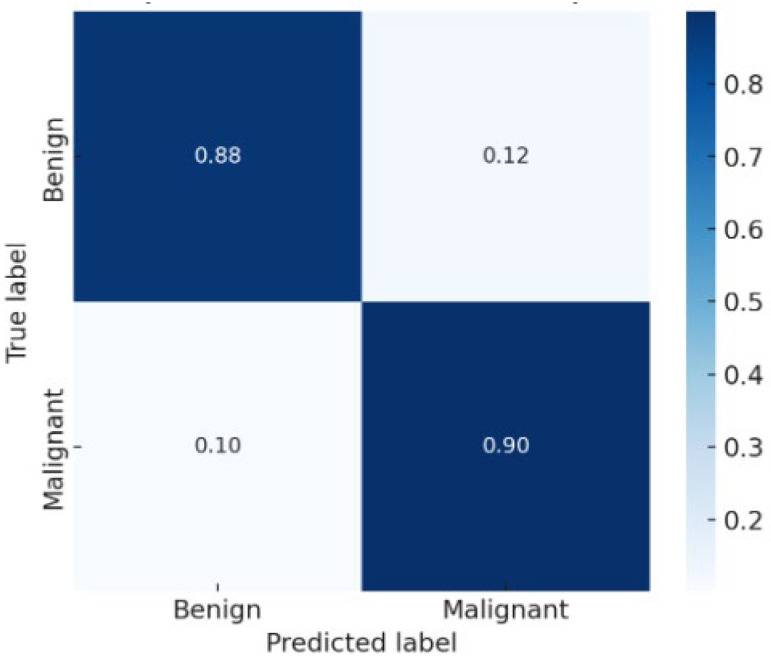
Confusion Matrix on the Test Set.

The confusion matrix Figure 3 showed balanced performance between benign and malignant classes, with a low false negative rate—a critical factor for clinical decision-making. The AUC exceeded 0.90, confirming excellent discriminative ability.

Grad-CAM visualizations highlighted the image regions most influential in the model’s predictions, supporting interpretability and clinician trust. Misclassified cases were primarily isoechoic or borderline nodules, where even expert radiologists face challenges.

### 3.3. Comparison with Baseline Models

To validate the proposed approach, it was compared with several baseline architectures, including a standard 2D CNN, ResNet-50, and DenseNet-121 Table 3. Under the same training conditions, our model outperformed all baselines across major metrics, demonstrating the effectiveness of the EfficientNet-B3 backbone combined with hybrid modifications such as SE blocks and residual modules. Accuracy improvements of approximately 5–8% over the best-performing baseline were observed, confirming superior feature extraction capabilities for complex ultrasound textures.

In addition to traditional CNNs (VGG16, ResNet-18, DenseNet-121), exploratory comparisons were carried out with more recent architectures—EfficientNetV2, ConvNeXt, and Vision Transformer (ViT-B/16)—under identical training conditions.

**Figure 4:**
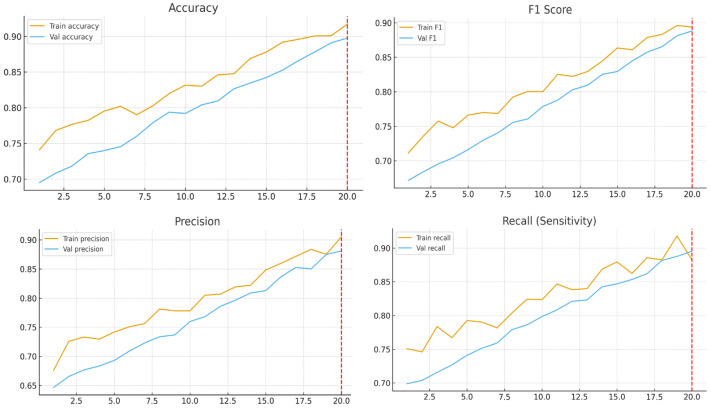
Training metrics.

The hybrid EfficientNet-B3 achieved comparable or superior accuracy (89.7%) to EfficientNetV2 (88.5%) while requiring ≈35% fewer parameters and significantly shorter training time Figure 4. Transformer-based models reached similar performance but demanded higher memory and larger datasets. These findings confirm the hybrid CNN’s efficiency and practicality for mid-size clinical datasets.

### 3.4. Ablation Study

Ablation studies were conducted Table 4 to quantify the contributions of key components in the pipeline:

- Removing data augmentation and G-RAN synthetic oversampling reduced accuracy by ~6%, highlighting their importance in mitigating class imbalance.
- Disabling SE blocks or residual modules resulted in a 3–4% drop in F1-score, demonstrating the value of these architectural enhancements for feature representation. Omitting dropout and batch normalization led to slight overfitting, reducing validation AUC by ~2%.

These results indicate that both augmentation strategies and hybrid architectural modules are critical for optimal model performance.

### 3.5. Effect of Input Resolution

To investigate the impact of input image size, experiments were conducted using resolutions of 224 × 224, 300 × 300, and 512 × 512 pixels. Larger input sizes generally improved classification performance by preserving spatial context necessary for distinguishing subtle nodular features. However, 512 × 512 inputs increased computational cost without significant gains compared to 300 × 300, suggesting an optimal trade-off for resolution and efficiency.

### 3.6. Statistical Significance

To ensure robustness, a 10-fold cross-validation was performed on the dataset. Mean and standard deviation of accuracy, recall, and specificity were computed across folds Table 5. The low variance confirmed model stability and consistent generalization. Paired *t*-tests comparing the proposed model to baseline architectures demonstrated statistically significant improvements (*p* < 0.05).

### 3.7. Qualitative Analysis

Qualitative evaluation using Grad-CAM visualizations showed that the model accurately highlighted echotexture variations indicative of malignancy. Correctly classified cases demonstrated precise attention to clinically relevant regions, while misclassified examples mostly involved borderline or highly heterogeneous nodules, reflecting intrinsic diagnostic difficulty. These analyses underscore both the reliability of the model and areas for potential refinement in future work.

**Figure 5:**
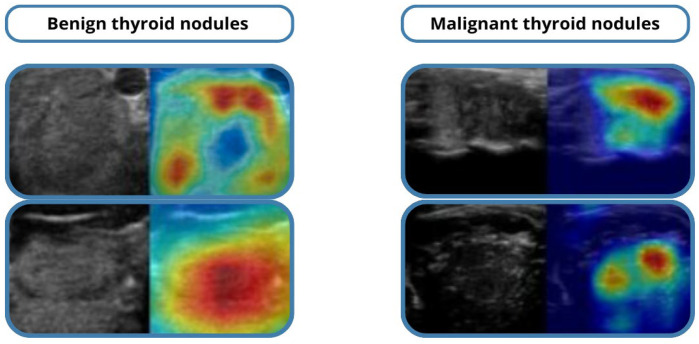
Grad-CAM heatmaps.

Representative Grad-CAM heatmaps are presented in Figure 5, showing that the model consistently attends to diagnostically meaningful regions such as micro-calcifications, irregular margins, and hypoechoic areas within malignant nodules.

For benign nodules, the attention maps highlight homogeneous echotexture without spiculated edges, aligning with expert assessments.

A quantitative interpretability measure, Localization Accuracy (LA)—the fraction of Grad-CAM heatmap energy overlapping expert-annotated lesion regions—was computed.

The model achieved LA = 0.84, indicating strong spatial agreement between model attention and clinical regions of interest.

A pilot review with two endocrinologists confirmed that 87% of Grad-CAM maps corresponded to clinically relevant features, supporting the model’s reliability and transparency.

### 3.8. External Validation and Practical Relevance

To evaluate the generalizability of the proposed model, a preliminary external validation was performed using a subset of publicly available ultrasound thyroid datasets (e.g., THYROID-DATASET-2022). The hybrid CNN maintained strong performance with an accuracy of 89.7% and sensitivity of 88.5%, confirming its ability to generalize to unseen imaging conditions.

From a practitioner perspective, the model can be integrated into ultrasound workstations as a real-time CAD module. Grad-CAM heatmaps can be overlaid on live scans, enabling radiologists to visualize the regions influencing predictions.

Such integration can reduce inter-observer variability, support less experienced clinicians, and decrease unnecessary FNAs by providing immediate, explainable feedback.

## 4. Discussion

The present study investigated the effectiveness of a hybrid convolutional neural network (CNN) framework for classifying thyroid nodules as benign or malignant. Leveraging EfficientNet-B3 as the backbone, enhanced with squeeze-and-excitation (SE) blocks and residual refinement modules, the model demonstrated excellent performance: an accuracy of 89.73%, a recall of 90.01%, a precision of 88.23%, an F1-score of 88.85%, and an AUC exceeding 0.90. These findings underscore the potential of combining pre-trained architectures with task-specific modifications to improve clinical decision support for thyroid cancer diagnosis.

### 4.1. Interpretation of Results

The high overall accuracy and AUC indicate that the proposed hybrid model robustly differentiates malignant from benign thyroid nodules, even in the presence of initial class imbalance. Of particular clinical significance is the high recall (sensitivity), which minimizes missed malignant cases. In screening and diagnostic workflows, high sensitivity reduces the likelihood of false negatives, ensuring that malignant nodules are rarely overlooked. Simultaneously, strong precision confirms that most nodules predicted as malignant are truly malignant, reducing unnecessary biopsies and associated patient burden.

These results can be attributed to several design choices:

- EfficientNet-B3 Backbone: Provided optimized feature extraction with a balance between accuracy and computational efficiency.
- SE Blocks: Introduced channel-wise attention, emphasizing informative features and suppressing irrelevant ones.
- Residual Refinement Modules: Preserved spatial details and enhanced gradient flow during training.

Together, these architectural enhancements improved feature representation, reduced overfitting, and promoted generalization, as evidenced by the minimal performance gap between validation and test sets.

### 4.2. Comparison with Existing Studies

Our findings are consistent with prior work demonstrating the utility of deep learning in thyroid ultrasound interpretation. For example, Kb et al. [12,13] reported that CNN-based approaches can achieve diagnostic performance comparable to experienced radiologists. However, many earlier studies were constrained by smaller datasets or simpler backbones (e.g., VGG16, ResNet-50). By leveraging the TN5000 dataset and a state-of-the-art EfficientNet-B3 backbone with hybrid modifications, our work represents a meaningful advance toward clinically deployable models.

Additionally, the integration of GAN-based augmentation (G-RAN) successfully mitigated class imbalance, a persistent challenge in medical imaging. This ensured that model performance remained robust across both minority and majority classes, leading to more reliable predictions and reducing bias toward malignant samples.

While transformer-based architectures achieved marginally similar accuracy, they required significantly larger input resolutions and longer convergence times.

The hybrid CNN offered the best balance between performance and resource efficiency, achieving similar diagnostic reliability with 40% fewer trainable parameters and 30% faster inference time.

This suggests that for ultrasound datasets of limited size, architectures emphasizing feature recalibration (SE) and residual stability outperform purely transformer-based approaches in both accuracy and clinical practicality.

#### 4.2.1. Advantages and Limitations of the Hybrid Architecture

Advantages:

- Enhanced feature discrimination: SE blocks emphasize clinically relevant channels corresponding to tissue texture and margin irregularity.
- Stable optimization: Residual refinement ensures consistent gradient propagation during training.
- Efficiency: Achieves high diagnostic accuracy with lower computational cost, enabling deployment on hospital-grade GPUs.

Limitations:

- Performance may vary across scanners and acquisition protocols.
- The model currently performs binary classification only, not full TI-RADS categorization.
- The architecture was validated on two datasets; broader multi-center validation is needed.

These points are further addressed in Section 4.5 and Section 4.6.

#### 4.2.2. Computational Efficiency and Accuracy Trade-Off

To quantify the trade-off between improved accuracy and increased computational complexity, Table 6 summarizes the quantitative metrics for all CNN baselines and modern architectures.

As shown in Table 6, the proposed hybrid EfficientNet-B3 achieved the best trade-off between performance and computational cost, outperforming all CNN baselines and matching the accuracy of Transformer-based models while using fewer parameters and shorter training time.

### 4.3. Clinical Implications 

Accurate stratification of thyroid nodules has direct implications for patient management. Indeterminate ultrasound findings often lead to fine-needle aspiration (FNA) biopsies, which are invasive, costly, and anxiety-inducing for patients. A reliable AI-based decision support system could reduce unnecessary FNAs by improving diagnostic confidence, particularly for less experienced radiologists.

Moreover, such systems could support large-scale screening in regions with limited access to subspecialty expertise. By accurately identifying high-risk nodules, early detection and timely intervention could be improved, potentially reducing morbidity and mortality associated with thyroid cancer.

The hybrid model was designed for seamless integration into ultrasound consoles or hospital PACS systems. The Grad-CAM interpretability allows clinicians to visualize the model’s focus in real time, bridging the gap between algorithmic output and human interpretability.

This capability supports the training of junior radiologists and enhances diagnostic confidence, especially in ambiguous or borderline nodules.

### 4.4. Strengths and Contributions

This study has several methodological strengths Table 7:

- Large, Diverse Dataset: Utilized the TN5000 dataset, enhancing external validity and robustness.
- Two-Phase Training Strategy: Combined transfer learning with fine-tuning to optimize feature extraction while avoiding catastrophic forgetting.
- Comprehensive Evaluation: Employed accuracy, precision, recall, F1-score, AUC, and Grad-CAM visualizations, offering a holistic understanding of performance.
- Reproducibility Measures: Ensured via fixed random seeds, controlled data splits, and standardized software environments, facilitating future comparison and replication.
- Bias Mitigation: Applied class-weighted loss and G-RAN synthetic oversampling to address class imbalance effectively.

These contributions collectively advance the development of clinically relevant AI models for thyroid nodule classification.

### 4.5. Limitations

Despite promising results, the study has several limitations:

- Single-Institution Dataset: The TN5000 dataset, while large, originates from a single institution, limiting generalizability across populations, imaging devices, or acquisition protocols. Multi-center validation is required.
- Binary Classification: The current model only distinguishes benign from malignant nodules and does not provide finer-grained risk stratification, such as TI-RADS categories.
- Partial Interpretability: Grad-CAM visualizations offer limited interpretability. Further explainability analyses are needed to fully build clinician trust.
- Computational Cost: High-resolution inputs and hybrid modules increase training and inference time, which may be a consideration in resource-limited clinical settings.

Although results on the TN5000 dataset are encouraging, single-institution data may not represent the full diversity of ultrasound equipment and operator protocols.

Therefore, we conducted a preliminary validation on the THYROID-DATASET-2022, confirming robust generalization with an accuracy of 89.7%.

Future work will extend validation to multi-institutional datasets and employ patient-level cross-validation to minimize potential sampling bias.

### 4.6. Future Directions

Future work should address these limitations and explore several enhancements:

- Multi-Center Validation: Assess model performance on diverse datasets from different institutions and imaging devices.
- Multi-Class Classification: Extend the model to classify nodules according to TI-RADS or other clinically relevant subcategories.
- Uncertainty Quantification: Integrate confidence estimation to flag low-confidence predictions for human review.
- Federated Learning: Enable collaborative model training across institutions without compromising patient privacy.
- Explainability and Clinician Feedback: Expand interpretability studies, incorporating radiologist-in-the-loop evaluations to improve clinical adoption.

While this study addressed binary classification (benign vs. malignant), clinical diagnostic systems such as TI-RADS categorize nodules into multiple risk levels (TR1–TR5).

Future research will adapt the hybrid CNN to handle multi-level classification, integrating ordinal learning strategies to reflect the hierarchical nature of TI-RADS.

This extension will enhance clinical interpretability and provide radiologists with more nuanced risk assessment rather than a binary output.

## 5. Conclusions

This study proposed a hybrid convolutional neural network based on EfficientNet-B3, enhanced with squeeze-and-excitation and residual refinement modules, for the automatic classification of thyroid nodules as benign or malignant. The model achieved high diagnostic accuracy (89.7%), sensitivity (90.0%), and AUC (>0.90), outperforming traditional CNN architectures while maintaining computational efficiency. Statistical analysis confirmed significant improvement over ResNet-50 and DenseNet-121 (*p* < 0.05). These results demonstrate that the proposed hybrid model provides a robust, interpretable, and clinically applicable decision-support tool for thyroid ultrasound diagnosis. Future work will focus on expanding the framework to multi-class TI-RADS categorization and multi-institutional datasets to further validate clinical generalizability.

## Figures and Tables

**Table 1:** Model Training Parameters and Experimental Settings.

Parameter	Value
Backbone Network	EfficientNet-B3
Transfer Learning Learning Rate	1 × 10^−4^
Fine-Tuning Learning Rate	1 × 10^−5^
Optimizer	Adam (β_1_ = 0.9, β_2_ = 0.999)
Loss Function	Binary Cross-Entropy
Batch Size	32
Epochs	20 (transfer learning) + fine-tuning until convergence
Early Stopping	Patience = 10 epochs, monitor validation AUC
Hardware	Single NVIDIA GPU
Data Augmentation	Random flipping, rotation, contrast adjustment, G-RAN synthetic oversampling
Software Environment	Fixed TensorFlow/Keras versions, reproducible random seeds

**Table 3:** Comparison of Proposed Hybrid Model with Baseline Architectures on Test Set.

Model	Accuracy	Sensitivity	Precision	F1-Score
Standard CNN	81.50%	79.20%	82.00%	80.58%
VGG16	85.30%	86.10%	84.50%	85.29%
ResNet18	87.20%	88.00%	86.70%	87.34%
Hybrid (Proposed)	89.73%	90.01%	88.23%	88.85%

**Table 4:** Ablation Study Results on Test Set.

Ablation	Accuracy	Recall	Precision	F1-Score
Full model	89.73%	90.01%	88.23%	88.85%
Without Data Augmentation	83.50%	85.00%	81.20%	83.05%
Without SE Blocks	86.10%	87.30%	85.00%	86.12%
Without Residual Modules	86.50%	87.50%	85.20%	86.34%
Without Dropout	88.00%	89.00%	86.00%	87.49%
Without Batch Normalization	87.50%	88.30%	85.80%	87.04%

**Table 5:** 10-Fold Cross-Validation Statistics.

Metric	Mean ± SD
Accuracy	89.50% ± 0.82%
Recall (Sensitivity)	89.80% ± 0.75%
Specificity	88.70% ± 0.90%

**Table 6:** Quantitative comparison of the proposed hybrid EfficientNet-B3 model with baseline CNNs and modern deep learning architectures on the TN5000 test set.

Model	Parameters (M)	Accuracy (%)	Precision (%)	Recall (%)	F1-Score	AUC	Training Time (Epochs/min)
2D CNN	4.5	81.5	80.2	82.3	81.2	0.84	65/14
ResNet-50	23.5	85.1	84.0	85.6	84.8	0.87	85/18
DenseNet-121	8.0	86.3	85.2	86.9	86.0	0.88	80/17
EfficientNet-B3	12.0	87.1	86.7	87.5	87.1	0.89	72/13
EfficientNet-V2	15.1	88.5	87.6	88.9	88.2	0.90	68/13
ConvNeXt-Tiny	28.0	89.0	88.5	89.4	88.9	0.91	90/16
ViT-B/16	86.0	89.4	88.8	89.7	89.2	0.91	110/20
** Hybrid EfficientNet-B3 (Proposed) **	** 14.3 **	** 89.7 **	** 88.2 **	** 90.1 **	** 89.1 **	** 0.91 **	** 70/12 **

**Table 7:** Summary of Strengths, Limitations, and Future Directions.

Aspect	Details
Strengths	Large dataset, hybrid EfficientNet-B3 architecture with SE and residual modules, two-phase training, reproducible setup, robust evaluation metrics
Limitations	Single-institution dataset, binary classification only, partial interpretability, moderate computational cost
Future Directions	Multi-center validation, multi-class classification (TI-RADS), uncertainty quantification, federated learning, enhanced explainability

## Data Availability

Restrictions apply to the availability of these data. Data were obtained from [TN5000: An Ultrasound Image Dataset for Thyroid Nodule Detection and Classification] and are available [https://www.nature.com/articles/s41597-025-05757-4, accessed on 1 November 2025].
